# Three-Dimensional Simulation Accuracy and Patient Satisfaction With Rhinoplasty

**DOI:** 10.1093/asjof/ojaf110

**Published:** 2025-09-09

**Authors:** Kosaku Yamamichi, Yuji Nakanishi, Chien Ying Chen

## Abstract

**Background:**

Achieving aesthetic satisfaction in patients undergoing rhinoplasty requires not only surgical precision but also effective communication of expected outcomes. Three-dimensional (3D) simulation has emerged as a promising tool for aligning patient expectations with surgical planning.

**Objectives:**

The authors of this study aim to evaluate the relationship between postoperative satisfaction in patients undergoing rhinoplasty and 3D simulation accuracy using the Crisalix simulation platform (Crisalix SA, Lausanne, Switzerland).

**Methods:**

This retrospective study included 38 patients who underwent aesthetic rhinoplasty with preoperative 3D simulation. Morphometric analysis compared the nasal parameters of the simulations with actual postoperative outcomes. Patient satisfaction was assessed using the FACE-Q scale. Similarity scores were rated by each patient and 2 independent surgeons, and the correlations were analyzed.

**Results:**

Crisalix simulation accuracy showed a strong correlation with postoperative patient satisfaction (*ρ* = 0.66, *P* < .001), second only to physician aesthetic scores (*ρ* = 0.71, *P* < .001). Subgroup analyses showed greater simulation discrepancies and lower satisfaction in revision cases, although the differences were not statistically significant.

**Conclusions:**

The authors of this study found higher predictive accuracy, utilizing 3D preoperative simulations, to be significantly associated with greater patient satisfaction. These findings underscore the potential utility of 3D simulation as a tool for aligning surgical outcomes with patient expectations and enhancing shared decision making.

**Level of Evidence: 4 (Diagnostic):**

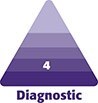

Aesthetic rhinoplasty is one of the most challenging procedures in cosmetic surgery. It significantly influences facial appearance and demands a high level of aesthetic satisfaction.^1-6^ Achieving patient satisfaction is a central goal for rhinoplasty surgeons. However, this is influenced not only by surgical techniques and morphological changes but also by the degree of alignment between the patient's preoperative expectations and the postoperative outcomes.^3^

Traditionally, surgical planning for rhinoplasty has been based on static photographs and manual examinations. However, this can lead to discrepancies between the perceived goals of the patient and the surgeon, potentially resulting in postoperative dissatisfaction.^7^ In recent years, preoperative simulation technology has rapidly evolved, and an increasing number of studies have evaluated its clinical utility.^8-15^ Among these, Crisalix (Crisalix SA, Lausanne, Switzerland) has gained attention as a cloud-based, user-friendly 3-dimensional (3D) simulation system with growing evidence supporting its utility in rhinoplasty.^12^

Despite the increasing research into the use of this technology in plastic surgery, there have been few quantitative assessments of the congruence between simulation outcomes and actual postoperative morphology or of the effects of such congruence on patient satisfaction.^10,13,16-18^ In particular, research with Asian patient populations has been limited. Moreover, to our knowledge, few studies have investigated the association between simulation accuracy, surgical technique, and morphological changes using a standardized patient-reported outcome measure (PROM) such as the FACE-Q.^3,13,15,16,19,20^

The authors of this study aimed to evaluate the relationship between postoperative satisfaction in rhinoplasty patients and the predictive accuracy of preoperative 3D simulation using the Crisalix platform.

## METHODS

### Patient Population

This retrospective study included patients who underwent aesthetic rhinoplasty at our institution between January 2022 and January 2024, all of whom viewed preoperative 3D simulations of the intended result on the Crisalix platform. Patients who underwent rhinoplasty for congenital deformities or posttraumatic indications were excluded.

Standardized photographs were taken preoperatively and at ≥6 months postoperatively from 3 views: frontal, right lateral, and left lateral. All images were captured by the same researcher using the same digital camera (NIKON Z6; Nikon Corporation, Tokyo, Japan) under standardized clinical lighting conditions. The same blue background was used for all photographs with uniform illumination. The images were saved in the JPEG format. The photographs were taken with the patient seated upright in a fixed chair. The camera was positioned 1.5 m from the patient at the level of their nose. The patient's head posture was adjusted so that the Frankfort horizontal plane (a line from the upper margin of the external auditory canal to the lower margin of the infraorbital rim) was parallel to the floor. Patients were instructed to gaze at a fixed point to ensure consistent visual orientation. The forehead and neck were fully visible in the photographs, with lips gently closed and a neutral facial expression. Eyeglasses were removed, and the eyes were fully open and horizontally aligned. To avoid facial rotation, the camera height was consistently matched to the level of the nose.

**Figure 1.**
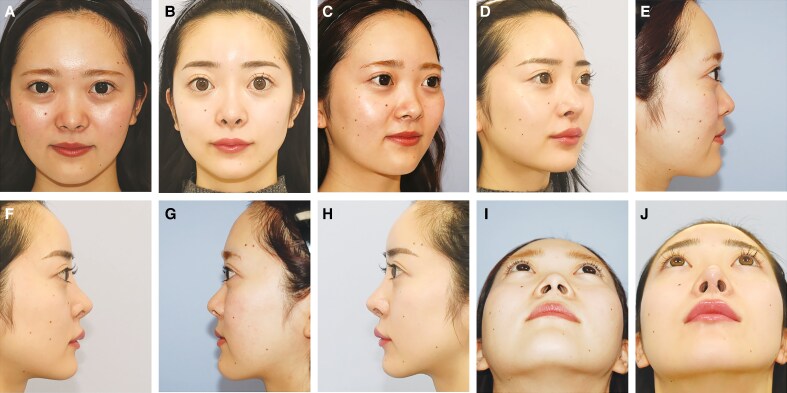
(A, C, E, G, I) Preoperative and (B, D, F, H, J) postoperative clinical photographs of a representative patient. The patient is a 22-year-old female who underwent dorsal augmentation with a silicone implant, septal extension graft using costal cartilage, and tip refinement with an auricular cartilage graft. The postoperative photograph was taken 6 months after surgery. (A) Frontal view (preoperative). (B) Frontal view (postoperative). (C) Right oblique view (preoperative). (D) Right oblique view (postoperative). (E) Right lateral view (preoperative). (F) Right lateral view (postoperative). (G) Left lateral view (preoperative). (H) Left lateral view (postoperative). (I) Basal view (preoperative). (J) Basal view (postoperative).

**Figure 2.**
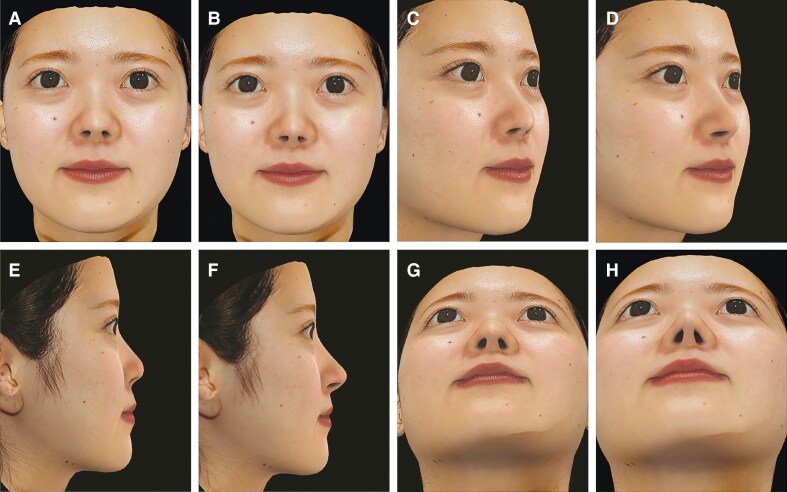
Three-dimensional (3D) images of a representative 22-year-old female patient using the Crisalix platform (Crisalix SA). (A, C, E, G) Preoperative 3D images and (B, D, F, H) simulated postoperative predictions were used to illustrate anticipated surgical outcomes and to evaluate morphological changes associated with rhinoplasty. (A) Frontal view (preoperative 3D image). (B) Frontal view (simulated postoperative prediction). (C) Right oblique view (preoperative 3D image). (D) Right oblique view (simulated postoperative prediction). (E) Right lateral view (preoperative 3D image). (F) Right lateral view (simulated postoperative prediction). (G) Basal view (preoperative 3D image). (H) Basal view (simulated postoperative prediction).

**Figure 3.**
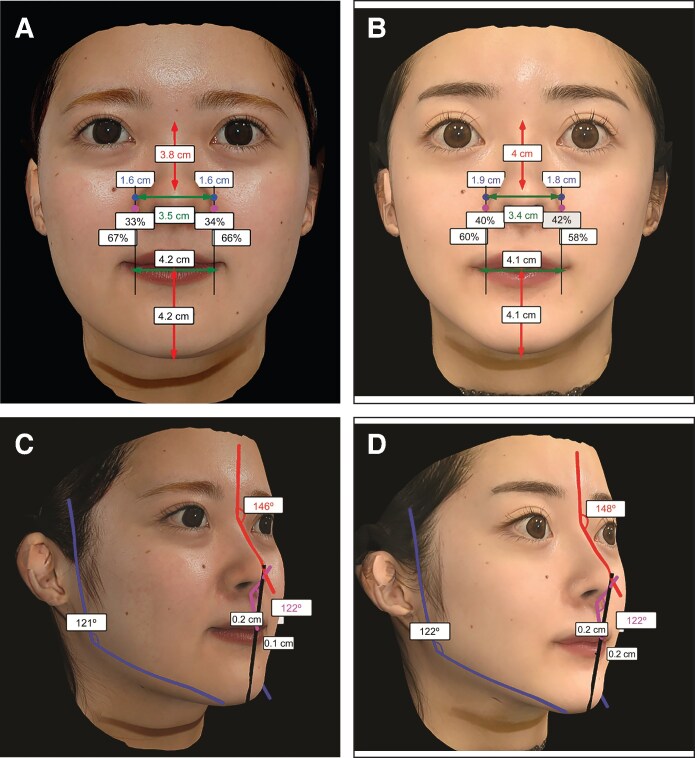
Morphological analysis of a representative 22-year-old female patient using 3-dimensional (3D) images from the Crisalix platform (Crisalix SA). (A, C) Preoperative 3D images derived from clinical photographs and (B, D) postoperative 3D images 6 months after surgery were analyzed to assess key nasal parameters. In A and B, red text indicates nasal length (NL), blue text indicates nasal tip projection (NTP; measured bilaterally), and green text indicates alar width (AW). In C and D, red text indicates the nasofrontal angle (NFA), and pink text indicates the nasolabial angle (NLA). The color coding refers to the text labels, not the arrows. (A) Frontal view (preoperative 3D image): analysis of NTP, NL, and AW. (B) Frontal view (postoperative 3D image): analysis of NTP, NL, and AW. (C) Right oblique view (preoperative 3D image): analysis of NFA and NLA. (D) Right oblique view (postoperative 3D image): analysis of NFA and NLA.

The captured images were uploaded to the Crisalix 3D simulation system (Platinum Face Plan; Crisalix SA, Lausanne, Switzerland), which automatically generates a 3D model of the face using the uploaded images. The system facilitates detailed facial analysis and provides morphological measurements, including linear distances, angles, and horizontal and vertical proportions. Representative measurement points are illustrated in Figures 1-3. In this study, 5 morphological parameters were assessed: nasal tip projection (NTP), nasal length (NL), alar width (AW), nasolabial angle (NLA), and nasofrontal angle (NFA). NTP was measured bilaterally from the alar base to the nasal tip, and the mean value of the right and left sides was used for analysis.

All procedures were performed either under general anesthesia or local anesthesia with intravenous sedation. The choice of anesthetic was based on the procedural complexity, the patient's preference, and the anesthesiologist's judgment.

### Patient Satisfaction

Patient satisfaction was evaluated preoperatively and postoperatively using the FACE-Q rhinoplasty module. The questionnaire includes the following subscales: satisfaction with nose, satisfaction with facial appearance, psychological function, social function, and satisfaction with decision.

Each item is scored on a 4-point Likert scale, ranging from 1 (“very dissatisfied” or “definitely disagree”) to 4 (“very satisfied” or “definitely agree”). Module scores were transformed using the Rasch model and ranged from 0 to 100. The primary satisfaction outcome was the patient's score on the satisfaction with nose subscale.

### Aesthetic Outcome Measures

Similarity scores were obtained from both patients and physicians. These represented the perceived resemblance between the preoperative 3D simulation and the actual nasal appearance at least 6 months after surgery. These ratings were collected in a private setting using a structured questionnaire. Patients and physicians were presented with standardized preoperative simulation images and postoperative photographs, which were displayed side by side to allow direct visual comparison. All responses were collected anonymously to ensure that the attending surgeon remained blinded to individual patient ratings. Ratings were given on a 4-point Likert scale, from 1 = not at all similar to 4 = very similar. They were subsequently converted to a 100-point scale (25, 50, 75, and 100) for statistical analysis.

Physician aesthetic scores were also collected. These were evaluations of the overall aesthetic outcome in each case. The scores were based solely on standardized postoperative photographs. To ensure that the evaluations reflected only the aesthetic quality of the postoperative appearance, no preoperative or simulation images were provided. The aesthetic assessments were performed by 2 independent board-certified plastic surgeons. Scores were given on a 4-point Likert scale and subsequently converted to a 100-point scale. The means of the 2 surgeons’ scores for each image were used for analysis.

### Statistical Analysis

All statistical analyses were conducted using R, v. 4.2.2 (R Foundation for Statistical Computing, Vienna, Austria). Continuous variables were expressed as mean ± standard deviation. Categorical variables were presented as counts and percentages (*n*, %). Non-normally distributed variables were presented as median and interquartile range. The Shapiro–Wilk test was used to assess the normality of continuous variables. For normally distributed data, paired or unpaired *t* tests were applied for within-group or between-group comparisons, respectively. For non-normally distributed paired variables, the Wilcoxon signed-rank test was used. Spearman's correlation coefficient was used to evaluate correlations between similarity scores, physician aesthetic scores, and patient satisfaction; *P*-values of <.05 were deemed to be statistically significant.

### Ethical Considerations

All procedures performed in this study were conducted in accordance with the ethical standards of our institutional research committee and the tenets of the 1964 Declaration of Helsinki and its later amendments. The study was approved by the IRB of Pearl Skin Clinic Tenjin (approval number: 20220104). The requirement for informed consent to participation was waived because of the retrospective nature of the study. Written consent to publication was obtained from the patient whose images are included in the figures.

## RESULTS

### Patient Demographics and Procedural Characteristics

A total of 38 patients who underwent aesthetic rhinoplasty with preoperative 3D simulation were included. The mean age was 31.8 (±15.3) years (range, 19-78 years), and the majority were female (*n* = 31, 81.6%). The mean follow-up period was 8.4 (±5.7) months (range, 6-36 months). Of the 38, 22 patients (57.9%) underwent primary rhinoplasty and 16 (42.1%) underwent revision procedures. The most commonly performed procedures were tip plasty (76%), conchal cartilage grafting (74%), columella strut grafting (58%), and dorsal augmentation (34%). Less common procedures included alar reduction (32%), nasal osteotomy (16%), dorsal hump reduction (13%), and removal of silicone implants (13%). The procedures are summarized in Table 1. No postoperative complications were observed in any of the patients.

**Table 1.** Patient Demographics and Procedural Variables

Characteristics	Value
Demographics	
Age (mean ± SD)	31.8 ± 15.3 (19-78)
Sex (F/M)	31/7
Follow-up period (mean ± SD)	8.4 ± 5.7 (6-36) months
Previous surgery	
None	22 (58%)
1 surgery	12 (32%)
2 surgeries	4 (11%)
3 surgeries	1 (3%)
Procedure type	
Tip plasty	29 (76%)
Conchal cartilage graft	28 (74%)
Columella strut graft	22 (58%)
Dorsal augmentation	13 (34%)
Alar reduction	12 (32%)
Silicone implant	12 (32%)
Septal extension graft	8 (21%)
Nasal osteotomy	6 (16%)
Dorsal hump reduction	5 (13%)
Columella correction	5 (13%)
Removal of silicone implant	5 (13%)
Dermal fat graft	5 (13%)
Removal of polycaprolactone implant	4 (11%)
Costal cartilage graft	4 (11%)

Summary of the patient demographics and surgical variables of 38 patients who underwent aesthetic rhinoplasty following preoperative 3-dimensional outcome simulation. Data are presented as mean (range), mean (±standard deviation [SD]), or number (%).

### Representative Case

A representative case is shown in Figure 1. The patient was a 22-year-old woman who underwent primary rhinoplasty. This procedure consisted of septal extension with costal cartilage, dorsal augmentation using a silicone implant, and tip refinement with conchal cartilage. Clinical photographs taken 6 months postoperatively showed a well-balanced nasal contour and improved tip projection and definition.

The morphological changes were observed preoperatively on the Crisalix simulation and confirmed postoperatively. These were increases in the NTP from 16 to 18.5 mm, in the NL from 38 to 40 mm, and in the NFA from 146° to 148°. The NLA remained unchanged at 122°.

Figure 2 shows the preoperative photographs of the patient and the simulated images illustrating the predicted changes based on the patient's aesthetic preferences and expected surgical outcomes. Figure 3 illustrates our morphological analysis of this case, with comparisons of the preoperative and postoperative angular and linear nasal parameters, including the NTP, NL, AW, NLA, and NFA, made using the Crisalix platform.

### Postoperative Morphometric Changes

A quantitative comparison of the preoperative and postoperative morphometric parameters in our cohort revealed statistically significant changes in NLs and NTPs. The mean NL increased from 41.2 (±2.2) to 42.0 (±1.8) mm (Δ = +0.7 [±1.6] mm, *P* = .010), and the mean NTP increased from 18.0 (±1.5) to 18.8 (±1.4) mm (Δ = +0.8 [±1.7] mm, *P* = .004). No significant changes were observed in the AW, NLA, and NFA (*P* = .632, .461, and .837, respectively; Table 2).

**Table 2.** Accuracy of Preoperative Rhinoplasty Outcome Simulations When Compared With Key Postoperative Morphological Changes in Nasal Parameters

Parameter	Pre Mean ± SD	Post Mean ± SD	Δ (Post–Pre)	*P*-value (Post vs Pre)	Sim Mean ± SD	Δ (Post–Sim)	*P*-value (Post vs Sim)
Nasal length (mm)	41.2 ± 2.2	41.9 ± 1.8	+0.7 ± 1.6	.01*	42.2 ± 2.0	−0.3 ± 1.7	.365
Nasal tip projection (mm)	18.0 ± 1.5	18.8 ± 1.4	+0.8 ± 1.7	.004*	19.5 ± 1.3	−0.7 ± 1.5	.009*
Alar width (mm)	35.8 ± 1.3	35.7 ± 1.3	−0.1 ± 1.0	.632	35.5 ± 1.2	+0.2 ± 1.0	.351
Nasolabial angle (°)	117.1 ± 5.6	116.6 ± 5.1	−0.5 ± 4.8	.461	115.9 ± 5.4	+0.8 ± 4.3	.32
Nasofrontal angle (°)	149.3 ± 3.6	149.4 ± 2.5	+0.1 ± 3.4	.837	147.8 ± 3.3	+1.7 ± 3.2	.005*

Comparison of the preoperative, postoperative, and simulated values for 5 key nasal parameters. Data are presented as means (±standard deviations [SDs]). Differences are shown as Δ (Post–Pre) and Δ (Post–Sim). The *P*-values were obtained using the Wilcoxon signed-rank test. *Represents statistically significant differences (*P* < .05).

### Comparison of Simulated and Postoperative Morphometry

The postoperative morphometric parameters were compared with the simulated values generated using Crisalix software to assess the simulation accuracy. Although the mean simulated NL (42.2 [±2.0] mm) was slightly greater than the mean postoperative NL (42.0 [±1.8] mm), the difference was not statistically significant (Δ = −0.3 [±1.7] mm, *P* = .365). However, the simulated and postoperative mean NTPs differed significantly (Δ = −0.7 [±1.5] mm, *P* = .009), indicating a consistent tendency in the simulations to overestimate projection. AW, NLA, and NFA did not differ significantly (*P* = .351, .320, and .005, respectively), as shown in Table 2.

### Patient-Reported Postoperative Outcomes

FACE-Q subscale scores demonstrated significant improvements in patient reported satisfaction after rhinoplasty, as summerized in Table 3).

- Satisfaction with the nose increased from a preoperative mean of 39.0 (±8.4) to 74.8 (±21.3) postoperatively (Δ = 35.9 [±23.7], *P* < .001).
- Satisfaction with facial appearance improved from 39.5 (±7.5) to 54.8 (±15.1) (Δ = 15.3 [±14.8], *P* < .001).
- Psychological function rose from 46.2 (±10.3) to 52.3 (±11.6) (Δ = 6.2 ± 9.7, *P* < .001).
- Social function increased from 48.1 (±12.7) to 55.2 (±15.1) (Δ = 7.1 [±10.7], *P* < .001).
- Satisfaction with the decision was assessed only postoperatively and had a mean score of 69.3 (±20.2).

**Table 3.** Patient-reported Satisfaction With Appearance and Related Factors on FACE-Q Subscales Before and After Aesthetic Rhinoplasty

Characteristics	Preoperative mean (SD)	Postoperative mean (SD)	Δ (Post–Pre)	*P*-value
FACE Q scale(range, 0-100)				
Satisfaction with nose	39.0 ± 8.4	74.8 ± 21.3	35.9 ± 23.7	<.001
Satisfaction with facial appearance	39.5 ± 7.5	54.8 ± 15.1	15.3 ± 14.8	<.001
Psychological function	46.2 ± 10.3	52.3 ± 11.6	6.2 ± 9.7	<.001
Social function	48.1 ± 12.7	55.2 ± 15.1	7.1 ± 10.7	<.001
Satisfaction with decision	—	69.3 ± 20.2	—	—

Patient satisfaction scores on FACE-Q subscales, including satisfaction with nose, facial appearance, psychological function, and social function. The table shows pre- and postoperative means (±standard deviations [SDs]) and the *P*-values obtained using the Wilcoxon signed-rank test.

All of the above *P*-values were derived using the Wilcoxon signed-rank test. There were significant improvements in patient-reported satisfaction and psychosocial functioning following aesthetic rhinoplasty.

### Aesthetic Outcome Measures and Correlation Analysis

The aesthetic evaluation results are summarized in Table 4. The mean patient similarity score was 72.4 (±16.2), whereas the mean physician similarity score was 70.4 (±18.7). The mean physician aesthetic score was 70.1 (±18.5). The mean postoperative FACE-Q satisfaction with nose score was 74.8 (±21.3). Correlation analysis with Spearman's *ρ* revealed the following results:

- Patient and physician similarity scores: *ρ* = 0.64. (*P* < .001)
- Patient similarity scores and physician aesthetic scores: *ρ* = 0.75 (*P* < .001)
- Patient similarity scores and FACE-Q satisfaction with nose scores: *ρ* = 0.66 (*P* < .001)
- Physician similarity scores and physician aesthetic scores: *ρ* = 0.67 (*P* < .001)
- Physician similarity scores and FACE-Q satisfaction with nose scores: *ρ* = 0.62 (*P* < .001)
- Physician aesthetic scores and FACE-Q satisfaction with nose scores: *ρ* = 0.71 (*P* < .001)

**Table 4.** Correlation Analysis of the Outcomes of Aesthetic Rhinoplasty and Preoperative 3-Dimensional Simulations

Characteristics	Value	*P*-value
Aesthetic score summary		
Patient similarity score	72.4 (16.2)	
Physician similarity score	70.4 (18.7)	
Physician Aesthetic Score	70.1 (18.5)	
FACE-Q satisfaction with nose	74.8 (21.3)	

Correlations between scores	Spearman's *ρ*	*P*-value
Patient similarity score vs physician similarity score	0.64	<.001
Patient similarity score vs physician aesthetic score	0.75	<.001
Patient similarity score vs FACE-Q satisfaction with nose	0.66	<.001
Physician Similarity Score vs physician Aesthetic Score	0.67	<.001
Physician similarity score vs FACE-Q satisfaction with nose	0.62	<.001
Physician aesthetic score vs FACE-Q satisfaction with nose	0.71	<.001

Descriptive statistics and Spearman's correlation coefficients for patient similarity scores, physician similarity scores, physician aesthetic scores, and FACE-Q satisfaction with nose scores. All correlations were statistically significant (*P* < .001).

Among these, the strongest correlation was observed between physician aesthetic scores and FACE-Q satisfaction with nose scores (*ρ* = 0.71), followed by patient similarity scores (*ρ* = 0.66).

### Comparison of Aesthetic Outcomes in Primary and Revision Rhinoplasty

A subgroup analysis was performed to compare aesthetic outcomes between primary and revision rhinoplasty cases. Although all measured scores—including patient and physician similarity scores, physician aesthetic scores, and postoperative FACE-Q satisfaction with nose scores—were lower in the revision group, none of the differences reached statistical significance. However, the difference between physician similarity scores approached significance (*P* = .051), suggesting a trend toward reduced simulation accuracy and aesthetic outcomes in revision cases (Supplemental Table 1).

## DISCUSSION

### 3D Simulation and Morphological Accuracy

In this study, the authors demonstrate that 3D simulation technology can serve as both a useful visual aid and a communication tool, bridging the gap between the aesthetic judgment of the surgeon and patient expectations. This is particularly critical in aesthetic rhinoplasty, which ranks among the most frequently performed cosmetic surgeries and demands a combination of surgical precision and meticulous preoperative planning to achieve optimal results.^21^

Understanding the diversity of patients’ aesthetic ideals and managing their expectations are crucial aspects of the planning process. As reported by Toriumi, differences in ethnic characteristics mean that rhinoplasty in Asian patients poses unique challenges that differ from those presented by white patients.^22^ Planning must be tailored to each individual's expectations, which can be challenging. Postoperative evaluation is heavily dependent on the patient's subjective satisfaction as well as objective morphological improvements. Therefore, bridging the perception gap between physicians and patients is vital to success.^7,15^ Recent advances in preoperative 3D simulation technology have garnered attention as a useful means of sharing treatment goals and building patient–physician consensus.

In this study, we quantitatively examined the predictive accuracy and clinical utility of the Crisalix 3D simulation system in rhinoplasty using the standardized PROM, FACE-Q.^3^ We analyzed the morphological congruence between simulations and postoperative measurements and subjective evaluations by physicians and patients. We also investigated the relationships between these parameters and patient postoperative satisfaction to elucidate the efficacy and limitations of Crisalix and its utility as a visual aid.

### Ethnic Morphology and Surgical Outcomes

Quantitative comparisons between preoperative and postoperative nasal morphology revealed significant increases in the mean NTP and NL. This is in concordance with the characteristic nasal morphology of East Asian patients, which includes a short NL, poor tip definition, a thick skin envelope, and weak tip support, as described in previous studies.^16^ Indeed, in our cohort, there was a high rate of tip plasty and conchal cartilage graft procedures, with many cases also undergoing dorsal augmentation. These surgical techniques aim to enhance NTP and extend the nasal septum, and the observed postoperative morphological changes were consistent with these surgical objectives.

Conversely, there was no statistically significant postoperative change in AWs. This may be attributed to the limited number of alar reduction procedures performed in this study. When we analyzed only the 12 patients who underwent alar reduction, there was a slight reduction in AW from a preoperative average of 34.9 (±2.8) mm to a postoperative average of 34.1 (±2.7) mm, but this did not reach statistical significance (*P* = .148). Further analysis of a larger number of cases is necessary to attain definitive conclusions.

Similarly, no clear postoperative changes were seen in the mean NLA and NFA. These angles are defined by multiple structures, including the nasal tip, columella, upper lip, and forehead, making them less susceptible to change through a single surgical technique. In particular, the NFA is heavily influenced by the bony structure of the nasal root and its relationship with the glabella.^23^ Thus, cases involving adjunctive procedures such as forehead fat grafting may exhibit different trends. The NLA is also influenced by multiple factors, including the presence of a columellar strut and the anteroposterior position of the upper lip, which can be affected by maxillary protrusion. Subgroup analyses of specific patients and procedures may reveal more pronounced changes.

Our morphological analyses identified significant increases in the mean NTP and NL, aligning with the typical aesthetic goals of rhinoplasty in East Asian patients.^17^ However, there have been few quantitative studies examining the relationship between such morphological changes and patient satisfaction.^10^

### Patient Satisfaction With Nasal Appearance and Related Outcomes

We compared the preoperative and postoperative FACE-Q scores of our cohort to elucidate the relationship between objective morphological changes and patients’ subjective satisfaction with outcomes. Our results showed statistically significant improvements across all patient-reported outcomes. Notably, satisfaction with the nose increased markedly from a preoperative average of 39.0 to a postoperative average of 74.8, a substantial rise of 35.9 points. Additionally, average satisfaction with facial appearance improved by over 15 points. These findings are consistent with those of Mookerjee et al.^13^ They suggest that rhinoplasty significantly impacts facial harmony and facial impression.

Moreover, moderate yet statistically significant improvements were observed in psychological function and social function, indicating that aesthetic rhinoplasty may contribute to enhanced self-perception and interpersonal confidence. This finding aligns with those of previous rhinoplasty studies using FACE-Q. Schwitzer et al reported significant postrhinoplasty gains in psychological well-being and social interaction in rhinoplasty patients.^19^ Satisfaction with decision, which we evaluated postoperatively, averaged 69.3 points, reflecting a relatively high level of patient satisfaction with the decision to undergo surgery.

Our findings demonstrate that aesthetic rhinoplasty can improve not only external appearance but also patients’ psychological and social quality of life. The FACE-Q provides a validated multidimensional framework for assessing surgical success from the patient's perspective, as demonstrated in previous studies.^3,19^

### Correlations Between Simulation and Satisfaction

Building on these results, we examined the correlations between aesthetic scores derived from simulations and the subjective evaluations of physicians and patients. The patient similarity score, indicating patient perceptions of congruence between simulations and postoperative nasal appearance, showed strong positive correlations with both the physician similarity score (*r* = 0.64, *P* < .001) and the physician aesthetic score (*r* = 0.75, *P* < .001). This indicates strong agreement between patients and physicians in the perception of similarity between simulations and actual outcomes. Those cases assessed as having high similarity were also perceived by physicians as having aesthetically favorable outcomes.

Moreover, both the patient and physician similarity scores were significantly correlated with the FACE-Q satisfaction with nose scores (*r* = 0.66 and *r* = 0.62, respectively; both *P* < .001). Although this suggests a relationship between visual congruence and postoperative satisfaction, it should not be interpreted as causal. Because the 3D simulations were customized to reflect each patient's desired aesthetic outcome, postoperative resemblance to the simulation may have reflected the degree to which the patient's expectations had been met. Hence, a close match between the simulation and the postoperative result likely signified that the patient's aesthetic goals had been achieved—thereby contributing to higher satisfaction with nose scores. Rather than the simulation directly influencing satisfaction, the alignment between desired and actual outcomes may be the more relevant factor. Additionally, the mean physician aesthetic score was highly correlated with patient-reported satisfaction (*r* = 0.71, *P* < .001), revealing a strong alignment between physicians’ aesthetic judgments and patients’ subjective evaluations. This study is among the few that have quantitatively explored the correlation between postoperative physician aesthetic evaluations and patient-reported satisfaction in aesthetic rhinoplasty. Although most previous research has focused on subjective patient outcomes or objective morphological analysis, few studies have assessed the relationship between expert aesthetic judgment and scores on validated PROMs, such as the FACE-Q.^3,15,19^ In this cohort, experienced surgeons used preoperative 3D simulation to visually align patient expectations and surgical goals. The resulting physician aesthetic scores may therefore reflect the shared aesthetic goals of the surgeon and patient, offering preliminary evidence in support of outcome assessment and shared decision making.^1,4,15^

### The Role of Shared Aesthetic Understanding

The strong correlation between physicians’ aesthetic evaluations and patient satisfaction observed in this study suggests that the aesthetic sensibility of experienced surgeons is closely aligned with patients’ perceptions of postoperative outcomes. Such aesthetic judgment reflects cultivated clinical intuition that is attuned to universally appealing forms and responsive to contemporary patient preferences across different cultural and racial backgrounds.^21^

Many patients undergoing rhinoplasty have difficulty grasping the gap between their aesthetic ideals and what is surgically achievable.^7,15^ Understanding postoperative changes in facial balance is not intuitive for most. Thus, integrating patients’ ideals of facial harmony and universally accepted beauty standards with surgeons’ aesthetic instincts rooted in clinical experience is an essential aspect of surgical planning. This integration must also be sensitive to prevailing trends and the cultural context.

In this study, Crisalix 3D simulation was used in all cases during preoperative counseling, enabling patients and surgeons to align their expectations regarding nasal morphology. This visual dialog can help to bridge perceptual gaps, foster mutual understanding, and improve patient satisfaction. Surgeons may propose nasal shapes based on widely accepted aesthetic principles; yet, these may not always reflect individual preferences. In such instances, 3D simulation functions as an effective communication tool, allowing detailed discussion of elements such as tip projection or nasal elongation and facilitating individualized planning.

Current simulation technologies are not without limitations and should be interpreted with discretion. Nevertheless, their utility in enhancing shared patient–doctor understanding and patient-centered outcomes in aesthetic rhinoplasty is evident. Our findings reinforce those of previous studies, highlighting the importance of shared aesthetic goals and the value of visual tools in optimizing postoperative satisfaction.^1,2,4^

### Simulation Accuracy and Technical Limitations

To further assess the morphological predictive accuracy of Crisalix simulations, we compared actual postoperative surgical measurements with the simulation predictions. Significant differences were observed in the mean NTPs and NFAs (*P* = .009 and *P* = .005, respectively). Specifically, the NTP was overestimated by an average of 0.7 mm, and the NFA was underestimated by an average of 1.7°.

The slight overestimation of NTP observed in the simulations may reflect intentional enhancement by surgeons to illustrate the expected postoperative outcomes more clearly to patients. Discrepancies between real and simulated outcomes can also result from factors such as soft tissue thickness and limitations in the structural support of the nasal septum. Nevertheless, the differences that we observed between simulated and actual surgical outcomes were minimal—<1 mm for NTP and <2° to 3° for the NFA—which are within clinically acceptable margins. Although the discrepancy in the NFA was statistically significant, its clinical relevance is limited. The results likely reflect the current limitations of 3D imaging technology in reproducing upper facial contours, particularly in the glabellar region. This is consistent with the observations of Pellitteri et al, who reported lower reproducibility of this region.^23^ The minor discrepancies between our simulations and the surgical outcomes can largely be attributed to user-related input variability or anatomical factors. Limitations in the software's predictive precision could also contribute to divergences between simulations and actual outcomes. These limitations should be recognized when interpreting simulation results. We found no statistically significant differences between simulated and actual NL, AW, or NLA, suggesting high reproducibility of these parameters. Overall, we found Crisalix 3D simulation to be a reliable tool for predicting linear and angular nasal morphology, particularly from the nasal tip to the dorsum. However, current 3D simulation technologies have inherent limitations in their ability to accurately represent postoperative outcomes. As emphasized by Rohrich and Ahmad, it should be clearly communicated during preoperative counseling that these simulations do not guarantee surgical results.^7^ A further limitation that should be borne in mind is the exclusively Asian patient cohort used in our study. This limits the generalizability of our findings to non-Asian populations because of anatomical and cultural differences between ethnicities.

### Challenges in Revision Rhinoplasty

Revision rhinoplasty is often more complex than primary procedures, particularly in Asian patients, in whom silicone implants and grafts are frequently used.^24^ These complexities can affect surgical planning and postoperative outcomes. Therefore, we conducted a comparative analysis between primary and revision cases to evaluate differences in outcomes and simulation accuracy. All of our evaluation indicators tended to be lower in the revision group than in the primary group, although no statistically significant differences were observed. However, the difference in physician similarity scores approached significance (*P* = .051), suggesting potential challenges in achieving morphological reproducibility and predictability in revision cases. FACE-Q subscale scores for satisfaction with nose and satisfaction with facial appearance were consistently lower in the revision group, with the difference in satisfaction with facial appearance nearing significance (*P* = .055). This trend may be attributable to several factors, including altered tissue characteristics, unexpected scar formation, and reduced elasticity in the skin and cartilage. These issues can pose technical challenges in revision rhinoplasty.^7,24^ In addition, when patients seek revision rhinoplasty, it is often because of dissatisfaction with their previous surgery. Therefore, unless the outcome of the revision exceeds their expectations, it may be difficult to achieve outcomes they consider satisfactory.^25^

It should also be noted that 3D simulations can contribute significantly to the expectations of revision patients. Simulations that closely match the patient's existing aesthetic ideals may lead to unrealistic or highly specific expectations. In such instances, there is an increased risk of patient dissatisfaction, particularly if there is disparity between the simulation and the surgical outcomes.^7^ Therefore, a thorough explanation of the limitations of visual simulations is of particular importance in revision cases. Surgeons should carefully align their expectations with those of their patients.

### Clinical Implications and Future Directions

Based on the findings of this study, the authors suggest that visual simulations can serve as a bridge between anticipated and achieved results in aesthetic rhinoplasty, for which personalized surgical planning is essential. Further validation and application development are anticipated. With ongoing advancements in artificial intelligence, 3D simulation technologies in aesthetic rhinoplasty are expected to evolve further, with improved predictive accuracy and clinical utility.

### Limitations

This study had several limitations. First, it was a retrospective, single-center study with a relatively small sample size (*n* = 38), which may limit the generalizability of our findings. The limited number of patients meant that subgroup analyses by gender or age were not feasible. To explore whether such demographic variables influence satisfaction or simulation congruence, further investigations are needed with larger cohorts. Second, although all postoperative assessments were conducted at least 6 months after surgery, variations in residual edema and scar tissue maturation may have affected the measured values slightly. Moreover, 6 months is a relatively short follow-up period for rhinoplasty, because stable final outcomes typically require at least 1 year. This should be kept in mind when interpreting our findings. Third, the physician aesthetic scores and simulation similarity scores were subjective evaluations by multiple raters. Although efforts were made to standardize the assessment process, complete elimination of interrater variability was not feasible. Fourth, current 3D simulation technologies are limited in the precision of their reconstructions. Thus, minor discrepancies between the simulated and real outcomes may result from imperfect image capture conditions, lighting, and rendering algorithms. Fifth, slight angular discrepancies, particularly in the lateral views, may have affected our comparative assessment. Although we endeavored to standardize the photographic conditions, it was challenging to capture perfectly matched lateral views because of slight variations in posture and head position. Finally, it should be noted that a surgeon's ability to achieve the simulated outcome varies significantly with experience and surgical skill. Preoperative simulation and imaging may serve as a useful tool; however, the findings of this study cannot be considered universally applicable for all practitioners. This is particularly true for younger and less experienced surgeons. Unless a surgeon can confidently create results corresponding to the preoperative simulation, they risk creating unrealistic expectations in the patient, potentially reducing patient satisfaction with outcomes.

## CONCLUSIONS

Our findings suggest that 3D simulation can serve as both a valuable tool for predicting postoperative morphology and as an effective means of promoting patient-centered care and shared decision making in aesthetic rhinoplasty. Because these visual communication tools continue to evolve, they are expected to further contribute to the realization of more precise and personalized surgical approaches.

## Supplemental Material

This article contains supplemental material located online at https://doi.org/10.1093/asjof/ojaf110.

## Supplementary Material

ojaf110_Supplementary_Data
