# Supplementary material for: Three-Dimensional Simulation Accuracy and Patient Satisfaction With Rhinoplasty
Source: Aesthet Surg J Open Forum. 2025 Sep 9;7:ojaf110. doi: 10.1093/asjof/ojaf110 (PMC12493035; doi:10.1093/asjof/ojaf110)
Supplement: ojaf110_Supplementary_Data [file ojaf110_supplementary_data.docx]

| **Supplementary Table 1.** Comparison of aesthetic outcomes between primary and revision rhinoplasty | | | |  |
| --- | --- | --- | --- | --- |
| **Characteristics** | **Primary (Mean ± SD)** | **Revision (Mean ± SD)** | **p-value** |  |
| Patient Similarity Score | 73.9 ± 14.4 | 70.3 ± 18.8 | 0.531 |  |
| Physician Similarity Score | 75.6 ± 16.6 | 63.3 ± 19.6 | 0.051 |  |
| Physician Aesthetic Score | 72.7 ± 17.5 | 66.4 ± 19.7 | 0.315 |  |
| postFACE-Q Satisfaction with Nose | 78.3 ± 20.6 | 70.1 ± 22.0 | 0.256 |  |
| postFACE-Q (Facial Appearance) | 58.5 ± 17.1 | 49.7 ± 10.3 | 0.055 |  |
| postFACE-Q (Psychological Function) | 53.5 ± 11.0 | 50.8 ± 12.6 | 0.488 |  |
| postFACE-Q (Social Function) | 57.4 ± 14.7 | 52.2 ± 15.6 | 0.304 |  |
| postFACE-Q (Satisfaction with Decision) | 72.9 ± 21.9 | 64.5 ± 17.0 | 0.194 |  |
| Independent t-tests were used to compare the mean (±SD) similarity scores, aesthetic ratings, and FACE-Q scale scores. | | | |  |
|  |  |  |  |  |
| SD, standard deviation  Data are presented as means (±standard deviations [SDs]). The P-values were obtained using the Wilcoxon signed-rank test. |  |  |  |  |
